# Depressive Symptoms and Risk of New Cardiovascular Events or Death in Patients with Myocardial Infarction: A Population-Based Longitudinal Study Examining Health Behaviors and Health Care Interventions

**DOI:** 10.1371/journal.pone.0074393

**Published:** 2013-09-25

**Authors:** Karen Kjær Larsen, Bo Christensen, Jens Søndergaard, Mogens Vestergaard

**Affiliations:** 1 Section for General Medical Practice, Department of Public Health, Aarhus University, Aarhus, Denmark; 2 Research Unit for General Practice, Department of Public Health, Aarhus University, Aarhus, Denmark; 3 Research Unit for General Practice, Department of Public Health, University of Southern Denmark, Odense, Denmark; S.G.Battista Hospital, Italy

## Abstract

**Background:**

Depressive symptoms is associated with adverse cardiovascular outcomes in patients with myocardial infarction (MI), but the underlying mechanisms are unclear and it remains unknown whether subgroups of patients are at a particularly high relative risk of adverse outcomes. We examined the risk of new cardiovascular events and/or death in patients with depressive symptoms following first-time MI taking into account other secondary preventive factors. We further explored whether we could identify subgroups of patients with a particularly high relative risk of adverse outcomes.

**Methods and Results:**

We conducted a prospective population-based cohort study of 897 patients discharged with first-time MI between 1 January 2009 and 31 December 2009, and followed up until 31 July 2012. Depressive symptoms were found in 18.6% using the Hospital Anxiety and Depression Scale (HADS-D≥8). A total of 239 new cardiovascular events, 95 deaths, and 288 composite events (239 new cardiovascular events and 49 deaths) occurred during 1,975 person-years of follow-up. Event-free survival was evaluated using Cox regression analysis. Compared to the 730 patients without depressive symptoms (HADS-D<8), the 167 patients with depressive symptoms (HADS-D≥8) had age- and sex-adjusted hazard ratios [HR] (95% confidence interval [CI]) of 1.53 (95% CI, 1.14–2.05) for a new cardiovascular event, 3.10 (95% CI, 2.04–4.71) for death and 1.77 (95% CI, 1.36–2.31) for a composite event. The associations were attenuated when adjusted for disease severity, comorbid conditions and physical inactivity; HR = 1.17 (95% CI, 0.85–1.61) for a new cardiovascular event, HR = 2.01 (95% CI, 1.28–3.16) for death, and HR = 1.33 (95% CI, 1.00–1.76) for a composite event. No subgroups of patients had a particularly high risk of adverse outcomes.

**Conclusions:**

Depressive symptoms following first-time MI was an independent prognostic risk factor for death, but not for new cardiovascular events. We found no subgroups of patients with a particularly high relative risk of adverse outcomes.

## Introduction

Major depression following myocardial infarction (MI) affects 16% to 27% of patients within 2 weeks after MI [1]. Post-MI depression is associated with about a doubling of the risk of new cardiovascular events or death [2], [3]. The explanatory mechanisms remain unclear [4] even if several mechanisms have been suggested, among others poor adherence to recommended lifestyle and secondary prophylactic medication advice [5], poor social support [6], [7], severe cardiac disease [8], low heart rate variability [9], inflammatory processes [10], and fewer invasive cardiovascular procedures [11]. In patients with stable coronary heart disease, Whooley et al. [12] found that the association between depressive symptoms and new cardiovascular events or death was largely explained by health behavior, especially physical inactivity. They therefore suggested that the adverse effect of depression on the prognosis of coronary heart disease might be prevented through behavioral modification. Exercise may be just as effective at reducing depressive symptoms in patients with coronary heart disease as antidepressants [13], but it is unknown whether these results apply to patients with MI [8], [12]. It also remains unknown whether subgroups of MI-patients with depressive symptoms are at a particularly high risk of adverse outcomes and whether treatment of post-MI depression improves the adverse cardiovascular outcomes in these persons. Zuidersma et al. [8] found that the association between post-MI depressive symptoms and new cardiovascular events or death was largely explained by cardiac disease severity, but they did not take into account physical activity, treatment of depression, or cardiac rehabilitation.

In the present prospective population-based cohort study of 897 participants, we examined the association between depressive symptoms following first-time MI and new cardiovascular events and/or death, taking into account disease severity, health behavior, use of health care interventions, and social and demographic characteristics. We further explored whether we could identify any subgroups of patients with a particularly high relative risk of adverse outcomes.

## Methods

### Ethic Statement

The study was approved by the Danish Data Protection Agency (J.nr. 2009-41-3018), the Scientific Research Evaluation Committee of the Danish Academy of General Practitioners (ref no. 03-2009), and written informed consent was obtained from the patients.

### Study Design and Participants

We conducted a population-based cohort study comprising people in the Central Denmark Region (1,250,000 inhabitants) with a first-time MI based on data from nationwide registers and questionnaires. The establishment of the cohort is described in detail elsewhere [14]. Briefly, we consecutively invited all patients discharged from hospital with a first-time MI from 1 January 2009 to 31 December 2009. Data on patients discharged with an MI (International Classification of Diseases (ICD-10) code I21) [15] were received from the Danish National Patient Register on a monthly basis. Patients who had been discharged with an MI between 1994 and 2008 were excluded to identify first-time cases. Information on name, address, and vital status was obtained from the Civil Registration System, which also provided the unique personal identification number used to link data between the registers and questionnaires.

### Data Collection

A questionnaire was mailed to all patients 12 to 14 weeks after their discharge from hospital. The questionnaire was pilot-tested and non-responders received two reminders [14].

### Depressive Symptoms

We assessed depressive symptoms using the Hospital Anxiety and Depression Scale (HADS) [16]. The HADS is a self-report instrument that consists of a depression scale (HADS-D) with seven items that are each answered on a four-point verbal rating scale with a score of 0–3 (total score 0–21). The HADS was designed to be valid in clinical populations with symptoms of physical disease. It thus avoids items that might be endorsed by physical rather than mental states [16]. The HADS has been validated in MI patients [17], [18] and has been proved to have satisfactory reliability (HADS-D Cronbach’s α ≈ 0.80) [17]. Compared with a physician-administered structured clinical interview for the Diagnostic and Statistical Manual of Mental Disorders, fourth edition (DSM-IV), which is the golden standard, a HADS-D≥8 identified possible cases of depression in a general practitioner population with a sensitivity of 80% and a specificity of 88% [19] and among MI patients with a sensitivity of 65% and a specificity of 90% [18].

### Co-morbidity and Cardiac Disease Severity

Information on co-morbidity was retrieved from the Danish National Patient Register, the Danish National Diabetes Register, and the prescription database covering the entire Central Denmark Region. The Danish National Patient Register provided information on stroke (ICD-10: I61, I63, I64), transient ischemic attack (ICD-10: DG45, DG46), heart failure (ICD-10: I11.0, I13.0, I13.2, I42.0, I42.6, I42.7, I42.9, I50.0, I50.1, I50.9), and revascularization (ICD-10: KFN, KFW) from 1994 to 2008. The Danish National Diabetes Register provided information on diabetes from 1990 to 2008 according to an algorithm developed on the basis of information from four nationwide registers [20]. The prescription database provided information on all reimbursed drugs according to the Anatomical Therapeutic Chemical Classification System (ATC), dispensing dates, and the total number of tablets dispensed. Persons were categorized with hypertension if they had redeemed a combination therapy with at least two antihypertensive drugs (ATC: C02A-C, C02D, C03A-E, C03X, C04, C05, C07, C08, C09) 0 to 180 days before the index MI, as validated previously [21]. Persons were categorized with depression before MI if they had redeemed an antidepressant (ATC: N06A) 0 to 180 days before the index MI.

The cardiac disease severity was measured using the British Medical Research Council (MRC) dyspnea score, a self-report instrument [22]. A score ≥3 has been shown to provide a simple and valid method for predicting overall mortality [23].

### Health Behavior, Health Status, and Health Care Interventions

Smoking, alcohol use, physical activity, intake of fruit and vegetables, intake of fish, intake of fish oil supplement, height, and weight (body-mass index  =  weight [kg] per height [m^2^]) were self-reported and classified according to the general recommendations from the Danish National Board of Health [14]. To assess physical activity, we asked, “How many days per week are you physically active for at least 30 minutes per day? Include any physical activity at your work or in your spare time where you feel that your pulse rate increases”. Participants chose from one of the following eight categories: no days, 1-6 days per week, every day. We defined cardiac rehabilitation [24] in the questionnaire and asked the patients whether they had participated in hospital-based phase II cardiac rehabilitation. Those who responded “yes, and I took part” were classified as “participants”; those who responded “yes, but I didn’t take part” or “no” were classified as “non-participants” [14].

Drug prescription data were obtained from the prescription database. Data on aspirin (ATC: B01AC06), clopidogrel (ATC: B01AC04), statins (ATC: C10AA), β-blockers (ATC: C07), ACE-inhibitors/angiotensin 2 receptor blockers (ATC: C09), and antidepressants (ATC: N06A) were collected. We calculated whether the patient had tablets available on the day that we sent the questionnaire (the number of tablets on the last redeemed prescription before the questionnaire was sent ≥ the number of days to the questionnaire was sent) and defined the patient as receiving treatment if tablets were available. We defined the patient as receiving secondary prophylactic medication if the patient was receiving treatment with three or more of the following drugs: aspirin, clopidogrel, statins, and β-blockers.

### Other Patient Characteristics

Data on age at MI and sex were obtained from the Civil Registration System. Each patient’s social and demographic characteristics from the year before MI (2008) were retrieved from the Danish Integrated Database for Labor Market Research [25].

### Cardiovascular Events and Death

Outcome events were new cardiovascular events (MI, heart failure, stroke, or transient ischemic attack), all-cause mortality, and a composite endpoint comprising a new cardiovascular event or death. Information on outcomes was collected from baseline to the last day of follow-up. The Danish National Patient Register provided information on cardiovascular events from all hospital contacts. Vital status (dead or alive) was obtained from the Civil Registration System.

### Statistical Analysis

Baseline differences in characteristics between MI patients with and without depressive symptoms were compared using t tests and χ2 tests. We calculated the event-free incidence-time as the time from 3 months after the MI (baseline evaluation of depressive symptoms) to the first cardiovascular event or death. If no event or death occurred, the patient was censored at 31 July 2012. Two persons emigrated during the time of follow-up and they were censored at the time of their emigration. Owing to the use of nationwide registers, we had complete follow-up of all patients. The risk of cardiovascular events or death associated with depressive symptoms was estimated using Cox proportional hazards models. We evaluated whether the hazard ratios (HR) of depressive symptoms following MI varied by subgroup by testing for interaction using Wald test in an age-adjusted model. The covariates for the multivariate model (age, sex, history of stroke, diabetes, or heart failure, cardiac disease severity, smoking, secondary prophylactic medication, and physical activity) were chosen on the basis of previous studies. No variable had more than 3.1% missing data. P<.05 was considered statistically significant.

## Results

Among a total of 1,288 eligible patients with first-time MI, 897 (69.6%) completed a questionnaire and 167 (18.6%) of those had depressive symptoms (HADS-D≥8). Non-responders were more often women, older, with fewer socioeconomic resources, and had more comorbid conditions than responders (Table S1). Compared with MI patients without depressive symptoms, MI patients with depressive symptoms had more severe cardiac disease, more comorbid conditions, used antidepressants more frequently, and received secondary prophylactic medication less frequently. They were also more likely to be female, living alone, outside the work force, smokers, less physically active, and have a smaller intake of fish than MI patients without depressive symptoms (Table 1).

**Table 1:** Baseline Characteristics of 897 Patients with First-time Myocardial Infarction, by Depressive Symptoms^1^

Characteristic			Depressive Symptoms(HADS≥8; n = 167)	No Depressive Symptoms(HADS<8; n = 730)	*P*
Social and demographic characteristics					
	Age, mean (SD), years		67.0 (12.2)	67.0 (11.5)	.967
	Sex, male, No. (%)		98 (58.7)	522 (71.5)	.001
	Marital status, living alone, No. (%)		72 (43.1)	211 (28.9)	<.001
	Education, No. (%)				
		<10 years	75 (45.7)	317 (45.0)	
		10-12 years	70 (42.7)	291 (41.3)	
		>12 years	19 (11.6)	97 (13.8)	.759
	Labor market status, No. (%)				
		Working	47 (28.1)	275 (37.7)	
		Retirement Pension	87 (52.1)	401 (54.9)	
		Out of the work force	33 (19.8)	54 (7.4)	<.001
Health status					
	Body Mass Index, mean (SD)		26.6 (5.4)	26.6 (4.5)	.974
Comorbid conditions					
	Hypertension		62 (37.1)	218 (29.9)	.068
	Stroke		16 (9.6)	39 (5.3)	.039
	Revascularization		24 (14.4)	59 (8.1)	.011
	Congestive heart failure		11 (6.6)	18 (2.5)	.007
	Diabetes mellitus		37 (22.2)	102 (14.0)	.008
	Depression		38 (22.8)	53 (7.3)	<.001
Cardiac disease severity					
	MRC dyspnea score ≥3, No. (%)		74 (44.6)	109 (15.0)	<.001
Medication use					
	Aspirin, No. (%)		129 (77.3)	576 (78.9)	.637
	Clopidogrel, No. (%)		124 (74.3)	566 (77.5)	.364
	β-blocker, No. (%)		137 (82.0)	588 (80.6)	.659
	Statin, No. (%)		139 (83.2)	608 (83.3)	.987
	ACE-inhibitor/AT-II-receptor block, No. (%)		92 (55.1)	341 (46.7)	.051
	Antidepressants, No. (%)		37 (22.2)	61 (8.4)	<.001
Potential behavioral mediators					
	Alcohol consumption >14/21 units per week, No. (%)		8 (4.8)	35 (4.8)	.995
	Smoking status, No. (%)				
		Current	43 (25.9)	136 (18.7)	
		Past	91 (54.8)	411 (56.5)	
		Never	32 (19.3)	181 (24.9)	.069
	Intake of fruit and vegetables ≥3 portions per day, No. (%)		52 (31.1)	278 (38.1)	.091
	Intake of fish ≥3 times per week, No. (%)		43 (25.8)	289 (39.8)	.001
	Intake of fish oil supplement, No. (%)		45 (27.0)	212 (29.1)	.582
Secondary prophylactic medication			110 (65.9)	538 (73.7)	.041
Physical activity, No. (%)					
	0 days per week		35 (21.1)	42 (5.8)	
	1-3 days per week		50 (30.1)	144 (19.8)	
	4-6 days per week		28 (16.9)	167 (22.9)	
	7 days per week		53 (31.9)	376 (51.6)	<.001
Participation in phase 2 cardiac rehabilitation			90 (54.6)	435 (59.7)	.227

Abbreviations: SF-12, Short-Form 12; MRC, Medical Research Council; ACE: angiotensin converting enzyme; AT: angiotension.

1 Totals may not sum to their respective totals due to missing data. No variable had more than 3.1% missing data.

**Figure 1:**
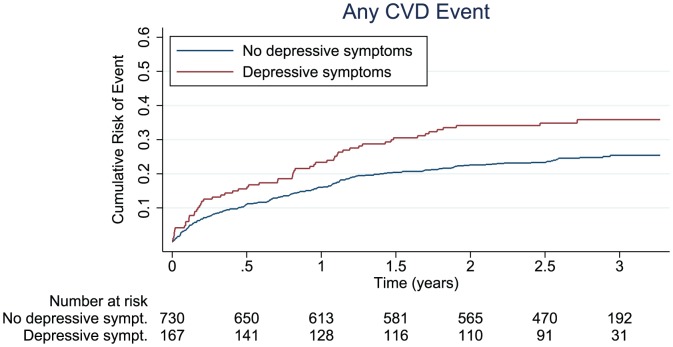
Cumulative Incidence of Any Event, Any Cardiovascular Event, or All-cause Mortality. For MI-patients with and without depressive symptoms, the Cumulative Incidence of Any Event was 47.2% and 30.0% (*P*<0.001), of Any Cardiovascular Event was 35.9% and 25.4% (*P* = 0.003) and of Death was 23.0% and 8.0% (*P*>0.001).

A total of 239 new cardiovascular events, 95 deaths, and 288 composite events (239 new cardiovascular events and 49 deaths) occurred during 1,975 person years of follow-up (mean follow-up: 2.2 years; SD 1.0); 59 (35.3%) new cardiovascular events, 37 (22.2%) deaths and 76 (45.5%) composite events occurred among persons with a HADS≥8, and 180 (24.7%) new cardiovascular events, 58 (8.0%) deaths and 212 (29.0%) composite events occurred among persons with a HADS<8 (Table 2). Compared to the MI-patients without depressive symptoms, the MI-patients with depressive symptoms had age- and sex-adjusted hazard ratios [HR] (95% confidence interval [CI]) of 1.53 (95% CI, 1.14–2.05; *P* = .005) for a new cardiovascular event, 3.10 (95% CI, 2.04–4.71; *P*<.001) for death and 1.77 (95% CI, 1.36–2.31; *P*<.001) for a composite event (Table 2, Figure 1). Adjustments for cardiac disease severity and comorbid conditions attenuated the HRs. The associations remained statistically significant for death (HR, 2.29; 95% CI, 1.48–3.53; *P*<0.001) and for a composite event (HR, 1.41; 95% CI, 1.07–1.86; *P* = .015) but not for a new cardiovascular event (HR, 1.25; 95% CI, 0.91–1.71; *P* = .164) (Table 3). Additional adjustment for physical activity further attenuated the associations. In the final adjusted model, MI patients with depressive symptoms had a 35% higher rate of a new cardiovascular event or death (HR, 1.35; 95% CI, 1.02–1.79; *P* = .034; Table 3) than MI patients without depressive symptoms. In the final model, depressive symptoms were associated with an increased rate of death (HR, 2.07; 95% CI, 1.32–3.25; P = .001), but not of new cardiovascular events (HR, 1.18; 95% CI, 0.86–1.62; *P* = .302; Table 3).

**Table 2:** Cardiovascular Events or Death During Follow-up, by Depressive Symptoms.

					Events, No. (%)			
Event					Depressive symptoms(HADS≥8;n = 167)	No Depressive Symptoms(HADS<8;n = 730)	Age- and Sex-Adjusted Hazard Ratio(95% Confidence Interval)	*P*
Any event					76 (45.5)	212 (29.0)	1.77 (1.36–2.31)	<.001
	Any CVD event				59 (35.3)	180 (24.7)	1.53 (1.14–2.05)	.005
			Heart failure		22 (13.2)	53 (7.3)	1.99 (1.21–3.29)	.007
			Stroke or transient ischemic attack		8 (4.8)	35 (4.8)	1.03 (0.48–2.23)	.936
			Myocardial Infarction		36 (21.6)	120 (16.4)	1.33 (0.91–1.93)	.140
	All-cause mortality				37 (22.2)	58 (8.0)	3.10 (2.04–4.71)	<.001

Abbreviation: CVD, cardiovascular disease.

**Table 3:** Association Between Baseline Depressive Symptoms and Subsequent Cardiovascular Events or Death.

	Hazard Ratio (95% Confidence Interval)		
Adjusted variables*	Any CVD event	Death	Any event
Age and sex	1.53 (1.143–2.05)	3.10 (2.04–4.71)	1.77 (1.36–2.31)
History of stroke, diabetes mellitus, or heart failure	1.39 (1.03–1.88)	3.01 (1.98–4.57)	1.65 (1.26–2.15)
MRC dyspnea score ≥3	1.25 (0.91–1.71)	2.29 (1.48–3.54)	1.41 (1.07–1.86)
Smoking status	1.24 (0.90–1.70)	2.28 (1.47–3.53)	1.421 (1.06–1.86)
Secondary prophylactic medication	1.24 (0.90–1.69)	2.23 (1.44–3.46)	1.40 (1.06–1.85)
Physical activity	1.17 (0.85–1.61)	2.01 (1.28–3.16)	1.33 (1.00–1.76)

Abbreviation: CVD, cardiovascular disease.

* Each model includes the variables from the preceding row so that the final model includes all the variables listed in this table.

**Figure 2:**
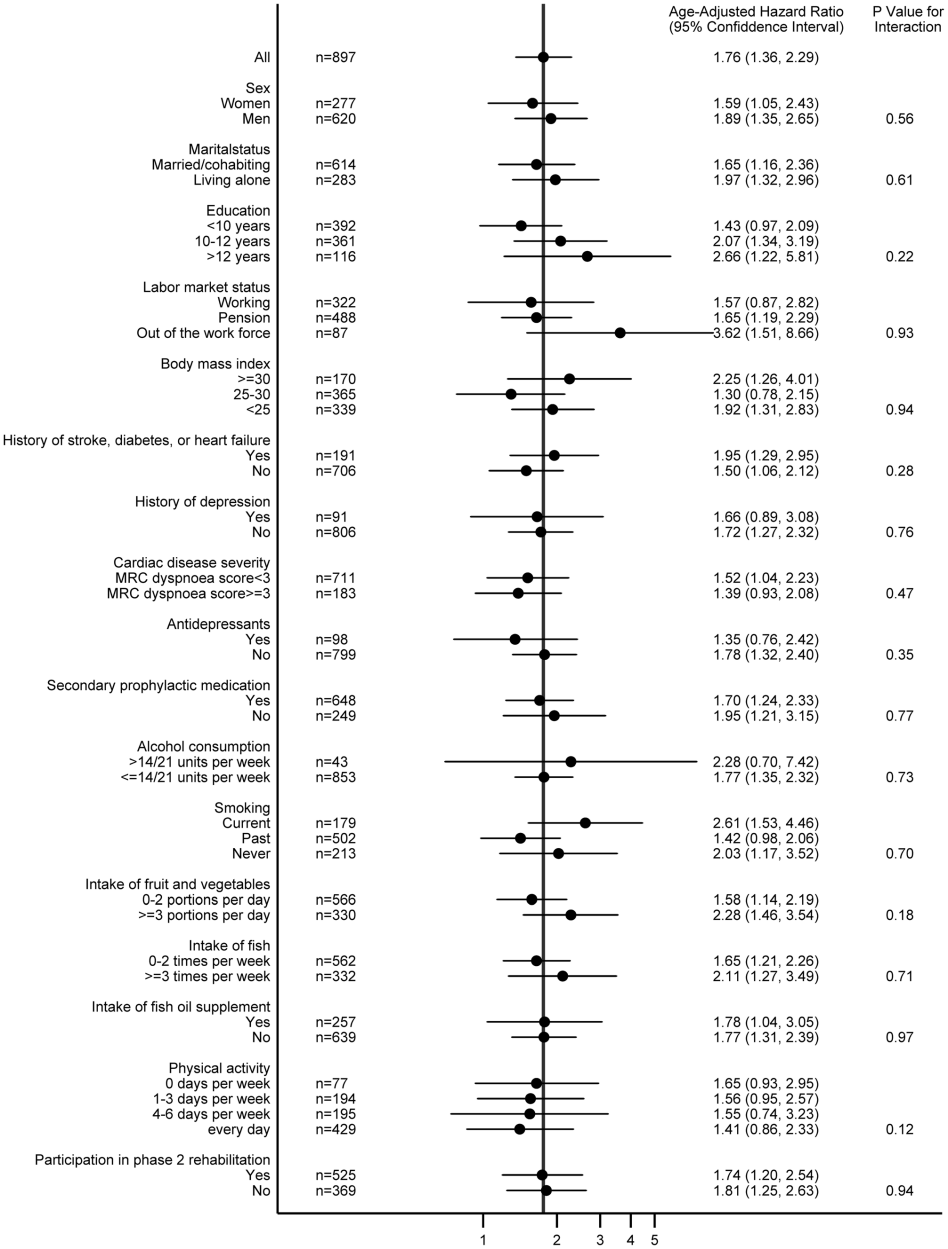
Association Between Depressive Symptoms and Subsequent Cardiovascular Events or Death for Patients with Myocardial Infarction by Specific Characteristics.

We found no statistically significant difference in the HR between subgroups of MI patients characterized by sex, marital status, education, labor market status, body mass index, comorbidity, history of depression, cardiac disease severity, antidepressant use, secondary prophylactic medication, alcohol consumption, smoking, intake of fruit and vegetables, intake of fish, intake of fish oil supplement, physical activity, or participation in phase 2 cardiac rehabilitation (Figure 2). However, the association between depressive symptoms and new cardiovascular events or death tended to be smaller among person who took antidepressants (*P* value for interaction = 0.35) or were physical active (*P* value for interaction = 0.12).

## Discussion

Depressive symptoms following MI was associated with an increased risk of a new cardiovascular event and/or death. However, the associations were confounded by the severity of the underlying heart disease and physical inactivity but not by other secondary preventive factors. After adjusting for these confounders, post-MI depressive symptoms remained an independent prognostic risk factor for death but not for new cardiovascular events.

A recent meta-analysis [2] found that patients with depressive symptoms following MI had a 2.25 (95% CI, 1.73–2.93) times higher risk of all-cause mortality and a 1.59 (95% CI, 1.37–1.85) times higher risk of new cardiac events than patients without such symptoms. Only eight studies [26]–[33] provided adjusted estimates and they were too heterogeneous to pool into a common estimate. In these studies, the estimates were on average 21% lower after adjustment for cardiac disease severity and comorbidity. In a recent study, Zuidersma et al. [8] found that one third to half of the association between post-MI depressive symptoms and cardiovascular events or death was explained by cardiac disease severity and previous MI. Whooley et al. [12] found that physical inactivity partly explains the association between depressive symptoms and new cardiovascular events or death in patients with stable coronary heart disease. Our study adds that it also seems to be the case for patients with first-time MI. These interpretations assume that physical inactivity act as a confounder (physical inactivity increases the risk of post-MI depression and increases the risk of adverse outcome) and therefore should be adjusted for. If physical inactivity act as a mediator (a step on the causal pathway from depressive symptoms to the adverse outcome) it should not be adjusted for [34]. In sub-analyses, we adjusted for other potential mediators (marital status, education, labor market status, body mass index, antidepressant use, and participation in phase-2 cardiac rehabilitation), but this did not change the estimates. In a sub-analysis, we also excluded patients with the more severe underlying physical disease (MRC≥3, previous stroke, heart failure or diabetes mellitus) and found that patients with depressive symptoms had a 1.91 (95% CI, 0.76–4.78; *P* = .166) times higher risk of death than patients without these symptoms, albeit the results did not reach statistical significance. These findings support that post-MI depressive symptoms seem to be an independent prognostic risk factor for death.

We examined the association between post-MI depressive symptoms and adverse outcome in several subgroups, but identified no factors that modified the risk. However, the sample size was low in some of the subgroups, and we found a tendency towards a lower association with increasing physical activity and among users of antidepressants. Larger studies are needed to clarify the impact of these potential modifiers of the association and to evaluate how such modifiers may be catered for in the treatment of post-MI patients with symptoms of depression. MI is a severe life event that even increases the risk of suicide [35] and general recommendations are that clinicians should recognize and treat post-MI depression [36]–[39]. Randomized trials have found that antidepressants and cognitive behavioral therapy reduce depressive symptoms in persons with MI [40], [41]. Physical exercise reduces depressive symptoms in patients with stable coronary heart disease [13], but no interventional studies have examined the effect of exercise on depressive symptoms in patients with a recent MI. In these patients, physical exercise should be supervised and preceded by a treadmill stress test for patient security and to reassure the patients that their hearts are strong enough to withstand regular exercise training [42]. Furthermore, it remains unknown whether treatment of depression improves the adverse cardiovascular outcomes of persons with coronary heart disease. Most trials have been underpowered to detect a potential effect on cardiovascular events or death, but one observational study has suggested that antidepressants reduce death and recurrent MI in patients with post-MI depression [43]. Mounting evidence shows that comprehensive and collaborative care is effective in managing persons with depression and co-existing physical illness [44], including MI [45]. Such care includes components like education about the condition, interventions to encourage physical exercise, and systematic monitoring of the patient’s adherence to medication [46]. Initiatives should be taken to implement comprehensive and collaborative care, and future studies should evaluate whether these strategies also improve the overall prognosis.

Previous studies suggested that the association between post-MI depressive symptoms depended on whether the depression was present before the onset of the MI or if it was a new depression that arose after MI [47], [48]. In a sub-analysis, patients with a history of depression were excluded, but this did not change the estimates.

The major strengths of our study were its population-based nature, the homogenous study population of patients with first-time MI, the high response rate, the complete follow-up, and the opportunity to analyze the importance of several suggested mechanisms. Our information on MI was registered prospectively and did not rely on participants’ or relatives’ memory. The MI diagnosis in the National Patient Register was based on current European Society of Cardiology criteria for MI, coded by the physician in charge of the discharge, and is known to have a high sensitivity (90%) and specificity (92%) that is unrelated to age [15]. The specificity of our study was even higher because we confirmed the MI diagnosis by reviewing the discharge summaries [14], which reduced the risk of information bias. A diagnosis of depression should ideally be based on a diagnostic interview. However, the diagnostic usefulness of a HADS≥8 has a high quality with a specificity of 90% in MI patients [18], and it hence performs well compared with a physician-administered structured clinical interview for DSM-IV. With a cut-off of a HADS-D≥11, the specificity approximates 100% [18], [19] and the use of this cut-off did not change the estimates of our analyses. Moreover, self-reported depressive symptoms are a more accurate predictor of cardiac morbidity and mortality than clinical depression [8]. HADS has a relatively low sensitivity (65%), probably because it does not include somatic symptoms of depression because they could be confused with symptoms of the cardiac disease. We may therefore have underestimated the number of patients with depressive symptoms leading to an attenuation of the association between depressive symptoms and new cardiac events or death. However, the prevalence rate (18.6%) of depressive symptoms in our sample corresponds to prevalence rates of other studies [1], [36]. We also reduced the risk of information bias by using previously translated and validated scales, pilot-testing the questionnaire among MI-patients, and using high-quality register data [15], [20], [25]. Comorbid conditions and social and demographic characteristics were evaluated before the MI. Even though we had a high response rate of 70%, we found that non-responders tended to be frailer than responders when we compared their social resources and comorbid conditions, which could affect our estimates. In order to address the potential risk of selection bias, we used antidepressant consumption as a proxy for depressive symptoms similarly to previous studies.[49] The estimates of the association between antidepressant consumption and new cardiovascular events or death in responders (HR, 1.49; 95% CI, 1.09–2.05) and in non-responders (HR, 1.40; 95% CI, 1.05–1.87) were similar, indicating that the possibility of selection bias was limited. Another strength is that we evaluated depressive symptoms 3 months post-MI in contrast to many other studies that evaluated depression during hospital admission. This allowed natural recovery of depressive symptoms after a stressful life event.

We were unable to evaluate whether the association is explained by potential biological mechanisms such as heart rate variability and inflammatory mechanisms because we had no information on this. In the study by Whooley et al. [12], inflammation as measured by C-reactive protein explained a small part of the association, but it is unclear whether inflammation acted as a mediator between depressive symptoms and cardiovascular events or death or as a marker of more severe disease. Our study evaluated depressive symptoms, cardiac disease severity, behavioral factors, and treatment strategies at the same time. We therefore cannot determine whether they were the cause or result of depressive symptoms. Lifestyle behavior was self-reported, and it is possible that patients with depressive symptoms were more likely to underreport adverse lifestyle including physical inactivity. However, patients with depressive symptoms in our study did report adverse lifestyle, and Whooley et al. [12] found no difference when substituting self-reported physical activity with an objective measure of physical fitness.

In conclusion, we found that depressive symptoms following first-time MI was associated with an increased risk of new cardiovascular events and/or death. The association was partly explained by disease severity and physical inactivity. Depressive symptoms remained an independent prognostic risk factor for death but not for new cardiovascular events. Mounting evidence shows that comprehensive and collaborative care is effective in managing persons with depression and co-existing physical illness [44], [50], including MI [45]. Such care includes components like education about the condition, interventions to encourage physical exercise, and systematic monitoring of the patient’s adherence to medication [46]. Our results support the relevancy of examining whether collaborative care also improves the overall prognosis of patients with depressive symptoms following MI.

## Supporting Information

Table S1 Comparison of responders and non-responders.
(DOCX)
