# Supplementary material for: Depressive Symptoms and Risk of New Cardiovascular Events or Death in Patients with Myocardial Infarction: A Population-Based Longitudinal Study Examining Health Behaviors and Health Care Interventions
Source: PLoS One. 2013 Sep 25;8(9):e74393. doi: 10.1371/journal.pone.0074393 (PMC3783427; doi:10.1371/journal.pone.0074393)
Supplement: Table S1 — Comparison of responders and non-responders. (DOCX) [file pone.0074393.s001.docx]

**Supporting Information**

| **Table S1.** Comparison of responders and non-responders | | | | | |
| --- | --- | --- | --- | --- | --- |
| **Variable** | | | **Responders**  **(n = 897)** | **Non-responders**  **(n = 391)** | ***P*** |
| Social and demographic characteristics | | |  |  |  |
|  | Age, mean (SD), years | | 67.0 (11.7) | 72.0 (14.9) | <.001 |
|  | Sex, male, No. (%) | | 620 (69.1) | 214 (54.7) | <.001 |
|  | Marital status, living alone, No. (%)* | | 283 (31.6) | 225 (57.5) | <.001 |
|  | Education, No. (%)* | |  |  |  |
|  |  | <10 years | 392 (45.1) | 194 (57.7) |  |
|  |  | 10-12 years | 361 (41.5) | 108 (32.1) |  |
|  |  | >12 years | 116 (13.4) | 34 (10.1) | <.001 |
|  | Labor market status, No. (%)* | |  |  |  |
|  |  | Working | 332 (35.9) | 75 (19.2) |  |
|  |  | Retirement Pension | 488 (54.4) | 266 (68.0) |  |
|  |  | Out of the work force | 87 (9.7) | 50 (12.8) | <.001 |
| Comorbid conditions† | | |  |  |  |
|  | Stroke | | 55 (6.1) | 39 (10.0) | .015 |
|  | Revascularization | | 83 (9.3) | 32 (8.2) | .536 |
|  | Congestive heart failure | | 29 (3.2) | 44 (11.3) | <.001 |
|  | Diabetes mellitus | | 139 (15.5) | 96 (24.6) | <.001 |
|  | Depression‡ | | 91 (10.1) | 87 (22.3) | <.001 |
